# Wastewater treatment using integrated anaerobic baffled reactor and Bio-rack wetland planted with *Phragmites* sp. and *Typha* sp.

**DOI:** 10.1186/s40201-014-0131-5

**Published:** 2014-10-25

**Authors:** Shervin Jamshidi, Abbas Akbarzadeh, Kwang-Sung Woo, Alireza Valipour

**Affiliations:** Department of Environmental Engineering, Faculty of Environment, University of Tehran, Tehran, Iran; Water Research Institute (WRI), Tehran, Iran; Department of Civil Engineering, Yeungnam University, Gyungsan, South Korea

**Keywords:** Anaerobic baffled reactor, Bio-rack wetland system, *Phragmites* sp, *Typha* sp, Domestic wastewater treatment

## Abstract

The purpose of this study is to examine the potential use of anaerobic baffled reactor (ABR) followed by Bio-rack wetland planted with *Phragmites* sp. and *Typha* sp. for treating domestic wastewater generated by small communities (751 mg COD/L, 500 SCOD mg/L, 348 mg BOD_5_/L). Two parallel laboratory-scale models showed that the process planted with *Phragmites* sp. and *Typha* sp. are capable of removing COD by 87% & 86%, SCOD by 90% & 88%, BOD_5_ by 93% & 92%, TSS by 88% & 86%, TN by 79% & 77%, PO_4_-P by 21% & 14% at an overall HRT of 21 (843 g COD/m^3^/day & 392 g BOD_5_/m^3^/day) and 27 (622 g COD/m^3^/day & 302 g BOD_5_/m^3^/day) hours, respectively. Microbial analysis indicated a high reduction in the MPN of total coliform and TVC as high as 99% at the outlet end of the processes. The vegetated system using *Phragmites* sp. showed significantly greater (p <0.05) pollutant removal efficiencies due to its extensive root and mass growth rate (p <0.05) of the plant compared to *Typha* sp. The *Phragmites* sp. indicated a higher relative growth rate (3.92%) than *Typha* sp. (0.90%). Microorganisms immobilized on the surface of the Bio-rack media (mean TVC: 2.33 × 10^7^ cfu/cm^2^) were isolated, identified and observed using scanning electron microscopy (SEM). This study illustrated that the present integrated processes could be an ideal approach for promoting a sustainable decentralization, however, *Phragmites* sp. would be more efficient rather than *Typha* sp.

## Background

Decentralized wastewater treatment systems (DEWATS) are appropriate for low-density communities and varying site conditions, and are more cost-effective than centralized practices. However, the efficiency of these systems depends on the attributes, and design criteria of the selected process [1]. Therefore, it is imperative to conduct research in order to develop a feasible and sustainable treatment technique for decentralization.

Anaerobic baffled reactor (ABR) reported to be a promising solution in domestic wastewater treatment [2–4]. The system recognized by incorporating the advantages of upflow anaerobic sludge blanket (UASB) and phase separation. It consists of alternating hanging and standing baffles, which compartmentalizes the reactor [5]. This practice leads the flow into intimate contact with the biomass [6,7], resulting in partial separation of acidogenesis and methanogenesis longitudinally down the reactor, and more stable operation [8–10]. It has been shown to provide higher resilience to hydraulic and organic shock loads, higher treatment rate, longer biomass retention times, and lower sludge yields than other anaerobic treatment systems [5]. Previous hydrodynamic studies revealed that the low dead space (7% to 30%) occurred in ABR [7]. Nasr et al. [2] reported removal efficiencies of 76% for total chemical oxygen demand (COD), and 55% for biochemical oxygen demand (BOD_5_) in ABR treating domestic wastewater at a hydraulic retention time (HRT) of 12 hours. Bodkhe [11] used a modified ABR in order to achieve 84% COD, and 87% BOD_5_ in treating municipal wastewater at a HRT of 6 hours. Feng et al. [12] used a bamboo carrier ABR to treat sewage achieving 69% COD reduction at a HRT of 18 hours. Despite the significant advantages and efforts made worldwide, typically the effluent of ABR technique is totally nutrients rich, oxygen deficit, and containing high concentration of unacceptable pathogen [2]. This would surely deteriorate the quality of surface waters in rural areas. In fact, it requires post treatment for removing the remaining organic matters, nutrients and pathogens.

In the recent years, the application of constructed wetlands for wastewater treatment has received greater attention since they offer a cost effective and an environmentally sound approach [13]. In that order, the possible applications of integrated anaerobic pretreated-constructed wetlands have been introduced as a promising model for DEWATS [14–16]. The major mechanism associated in constructed wetland for pollutant removal is the interaction between the bacterial metabolism and plant uptake, respectively, termed as rhizoremediation and phytoextraction [17]. Plants transfer oxygen to their root system and release a fraction of this oxygen into the rhizosphere for aerobic bacterial activity [18]. The type of aquatic plant species can also act as an important biotic factor in the treatment efficiency of constructed wetlands [19]. In that order, among various aquatic weeds, *Phragmites* sp. and *Typha* sp. are two well-known macrophytes demonstrated as potentially valuable vegetation in the natural purification process [20]. Nevertheless, the use of constructed wetlands, either as a standalone or combined solution, could be restricted by choking, clogging, slow mass transfer, poor root penetration into the multilayer soil column, high area requirement and capital investment. It is implied that conventional decentralized treatment processes are required to be optimized and upgraded to prevail over the limitations.

In our earlier recent work, the application of ABR has been studied comprehensively to optimize its design and operation in pollutant removal from domestic wastewater (~350-750 mg COD/L), and it was simulated by artificial neural network [9,10]. On the other hand, the Bio-rack system (BRS) is one of the latest evolutions of wetland technologies that developed to overcome the drawbacks of conventional wetland processes [21]. This is a robust biological practice that can be applied to the efficient and reliable elimination of pollutants, even under unfavorable influent conditions stressed by high total dissolved solids and heavy metal [21,22]. Analytical data which collected during the treatment of domestic wastewater (COD ≤440 mg/L, BOD_5_ ≤ 238 mg/L) showed that this process able to reduce up to 80% COD, and 90% BOD_5_ at a HRT of 10 hours [21]. The process is designed by incorporating the advantages of engineered attached growth (using a support matrix of the Bio-racks) and phytoremediation systems. The Bio-rack technique provides a desirable condition for plant root growth, efficient oxygen diffusion, and a distinguished surface area for a superior microbial population responsible for organic degradation at much low area requirement and capital cost [23]. The interrelationships between the plants and microorganisms associated with biofilms attached to the racks, stems, roots, and water column form a high rate wetland system. A further study also revealed the efficiency of this method planted with *Thalia dealbata*, *Acorus calamus*, *Zizania latifolia* and *Iris sibirica* for river water treatment [24,25]. Marchand *et al*. [26] successfully applied a Bio-racks filled with a homogeneous mix of gravels and perlite for the removal of copper ion from synthetic Cu-contaminated wastewaters using *Phragmites australis*, *Juncus articulatus* and *Phalaris arundinacea*.

Hence, it is recommended that using Bio-rack wetland upgraded with ABR should be considered as a novel scientific advancement and integrated approach. This innovation has not been documented before; its performance would be controversial and requires to be optimized thoroughly. This study is aimed to develop and examine the performance of an integrated process of ABR and BRS for effective treatment of domestic wastewater which could be implemented in decentralization practices. The potential ability of two aquatic plants, included *Phragmites sp*. and *Typha sp*., in wetland phytoremediation were also compared to find the most efficient and well matched vegetation in the combined ABR and BRS. This is important to have a comparative assessment on the use of aquatic weeds in the phytoremediation systems, where the most suitable macrophytes are available.

## Materials and methods

### The characteristic of wastewater

The overall characteristic of domestic wastewater during the study period was as follows: pH 7.44 ± 0.12, total chemical oxygen demand (COD) 751 ± 43 mg/L, soluble chemical oxygen demand (SCOD) 500 ± 45 mg/L, biochemical oxygen demand (BOD_5_) 348 ± 26 mg/L, dissolved oxygen (DO) 0.19 ± 0.07 mg/L, total suspended solids (TSS) 528 ± 41 mg/L, total nitrogen (TN) 64.65 ± 5.14 mg/L, phosphate (PO_4_-P) 13.7 ± 1 mg/L, total coliform 5.27 × 10^7^ ± 1.8 × 10^7^ MPN/100ml, and total viable count (TVC) 3.30 × 10^7^ ± 6.53 × 10^6^cfu/ml.

### Chemicals and instrumentation

The chemicals used in this study were of analytical grade (from Merck, Germany) as follows: Borax, Bromocresol green (5%), Citric acid, Ferroin indicator, Formaldehyde solution, Methyl red (1%), Starch, Ag_2_SO_4_, CaCl_2_, C_20_H_14_O_4_, C_2_H_5_OH, CuSO_4_.5H_2_O, FeCl_3_.6H_2_O, Fe(NH_4_)_2_(SO_4_)_2_.6H_2_O, H_3_BO_3_, HCl, HgSO_4_, H_2_SO_4_, K_2_Cr_2_O_7_, KI, KH_2_PO_4_, K_2_HPO_4_, K_2_SO_4_, MnSO_4_.4H_2_O, NaCl, NaHCO_3_, NaN_3_, NaOH, Na_2_SO_4_, Na_2_S_2_O_3_.5H_2_O, Na_2_HPO_4_.7H_2_O, Na_2_S_2_O_3_.5H_2_O, (NH_4_)_6_Mo_7_O_24_.4H_2_O, (NH_4_)_2_S_2_O_8_, NH_4_Cl, MgSO_4_.7H_2_O, Nutrient agar, and MacConkey broth.

The sample pH was measured by a pH-meter model 75P (Istek, Korea). COD tests were performed by using the open-reflux digestor model EME6/CEB (Electrothermal, UK). TN was examined via the semimicro Kjeldahl setup. UV-visible spectrophotometer model P/N BUV40X00 (PerkinElmer, USA) was used to measure PO_4_-P. Steroscan 440 scanning electron microscope (SEM, Leica, Cambridge, UK) was used during observation of active biomass on the support matrix (Bio-rack).

### Pilot plant setup

**Figure 1:**
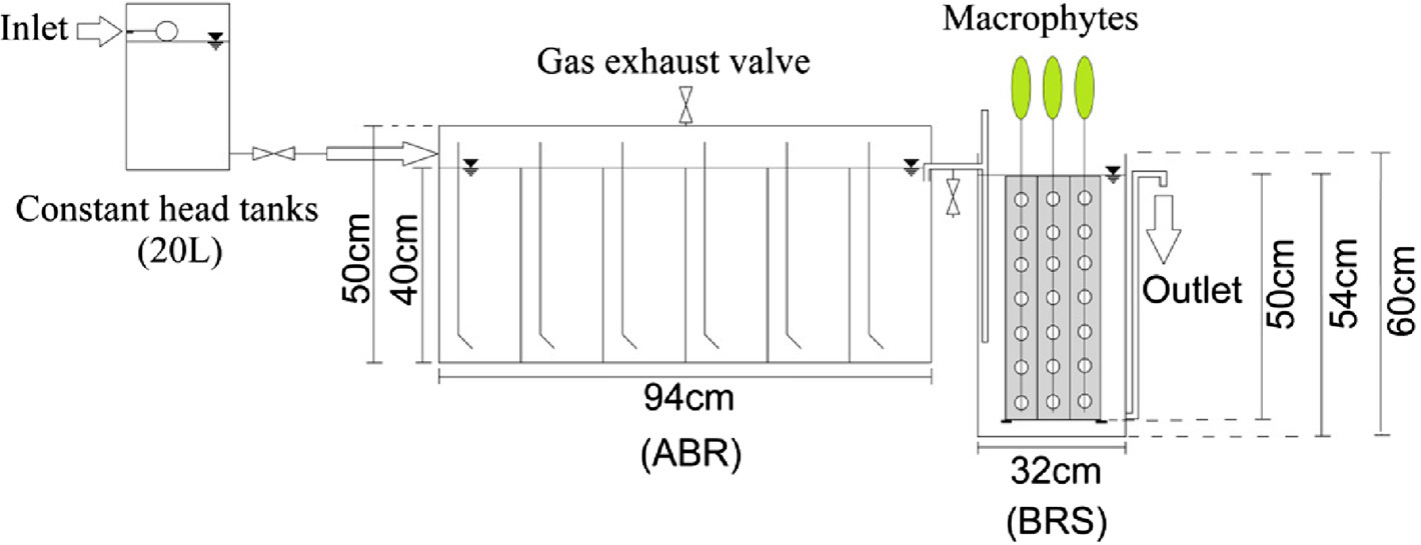
**Schematic diagram of the integrated treatment systems** **(** **ABR** **:** **anaerobic baffled reactor** **(** **ABR** **),** **BRS** **:** **bio** **-** **rack system** **).**

Two bench scale anaerobic baffled reactors (ABR) in parallel followed by horizontal flow Bio-rack system (BRS) vegetated with *Phragmites* sp. and *Typha* sp. were set-up on the premises of Water and Wastewater Research Centre (WWRC), Water Research Institute (WRI), Tehran, Iran. The pilot plants were comprised of three major sections including storage tanks (70 L), constant head tanks (20 L), and wetland units (Figure 1).

Each Plexiglas anaerobic baffled reactor (Length: 0.94 m, Width: 0.16 m, Height: 0.5 m) with 60 L net volume, consists of equally six compartments. The compartments were arranged by further vertical baffles dividing upflow-to-downflow parts by a ratio of 3:1 to affect the fluid dynamics in the sludge bed [7]. In this process, the wastewater force to flow under and over the baffles from one compartment to the next as it passes from the inlet to the outlet. The biogas formed during the anaerobic fermentation was released by the exhaust valves located on the top of the reactor.

The wetland systems (Bio-rack units) included a rectangular tank measuring 0.32 m × 0.3 m × 0.6 m (L × W × H), an effective depth of 0.54 m, and a 50 L void volume in the presence of vegetation and other internals. Unique feature of this system is the presence of 15 vertical PVC pipes (a 500 mm effective height × 60 mm outer diameter), free of soil strata, assembled as a rack and called Bio-rack. The pipes contained numerous surface perforations (20 mm diameter) for liquid transportation. These Bio-racks are involved in the treatment process by holding vegetation and providing an abundant attachment site for microbial growth. In this practice, the wastewater is distributed uniformly and then flows horizontally through the support matrix (Bio-racks), stems and roots of the plants to treat by physical and biological processes. The occupied area and specific surface of the matrix Bio-rack were 0.06 m^2^ and 55 m^2^/m^3^ effective volume (57 m^2^/m^3^ filled volume), respectively. Sixty *Phragmites* sp. and thirty *Typha* sp. plants were held in the support matrix of the Bio-rack at the initial stage of the plantation. The lower number of *Typha* sp. was used because these plans are larger in size compared to *Phragmites* sp. The inlet and outlet arrangements were made as per standard practices.

### Start-up and acclimatization

The experiments were initiated with the batch mode of merely anaerobic baffled reactors by seeding the mixture of cow dung and sewage sludge at the ratio of 1:2 (50% v/V) on effluent wastewater in each compartment for 2 weeks. The content of the reactors was recycled for homogeneity in the same period. Subsequently, the two comparable anaerobic baffled reactors in combination with Bio-rack systems were operated through serial exposure of sewage starting from 25% loading rate, which was increased gradually to 100% at an overall HRT of 44 hours over a 25 day period. In this stage, the systems continued to perform for more 45 days until the reduction in the percentage of COD reached a constant level, which was considered as the acclimatization period. Initially, the plant density was low and gradually the plant growth and reached their maximum possible number as bacterial growth was enhanced. In that order, the ratio of the volatile fatty acids to total alkalinity concentration was observed to be maintained below 0.3 at the outlet end of the ABR during studies.

It is noteworthy to say that the *Phragmites* sp. and *Typha* sp. were obtained from marshy lands of Tehran Province and Malayer County, respectively. Sampling was accomplished with simple hand tools. The underground parts (roots and bulbs) of the plants were carefully dug up, and the soil removed with care. A plastic collection container containing water marsh was used for transferring the sampled plants and minimizing their damage through several mechanisms. In the lab, the roots of sampled plants were washed with tap water to remove any remaining adsorbed soil particles before preliminary plantation. The type of the plants determined with the help of the National Botanical Garden, Teharan, Iran.

### Operational procedure

The integrated systems of ABR and Bio-rack wetland vegetated with *Phragmites* sp. and *Typha* sp. were operated in an ambient air temperature (25-29°C), where sunlight could effectively penetrate. Temperatures were not controlled except to prevent the effect of cold weather. After the acclimatization period, the experiments were initiated during February 2012 to 2013 to determine the state of present processes denoted by hydraulic retention time (HRT), treatment performance and plant growth with the intention of organic pollutant elimination considering the effluent discharge norms specified by the national pollution control board of Iran (COD <100 mg/L, and BOD_5_ < 30 mg/L), that is required for discharge into natural water resources. Under steady state condition, the systems were operated in six phases (Table 1). The daily flow was calculated by taking into account the theoretical HRT of ABR, and subsequently examined the HRT of wetland unit through each phase. The theoretical HRT, t (hours), of each treatment unit was established from the following equation:

**Table 1:** **Experimental conditions implied in wastewater treatment**

**Phase**	**I**	**II**	**III**	**IV**	**V**	**VI**
**Item**						
Operation period (day)	60	60	60	60	60	60
Overall HRT (hours)	41	33	27	24	21	18
HRT_ABR_ (hours)	22	18	15	13	11	10
HRT_BRS_ (hours)	19	15	12	11	9	8
Q (L/day)	65	80	96	114	126	144
OLR						
(g COD/day)	50 ± 2.63	61 ± 3.02	68 ± 2.02	85 ± 6.02	93 ± 6.46	113 ± 2.56
(g BOD_5_/day)	23 ± 1.96	29 ± 2.60	33 ± 2.09	40 ± 3.51	43 ± 3.07	50 ± 4.11
(g COD/m^3^/day)^*^	455 ± 24	553 ± 27	622 ± 18	769 ± 55	843 ± 59	1024 ± 23
(g BOD_5_/m^3^/day)^*^	205 ± 18	260 ± 24	302 ± 19	361 ± 32	392 ± 28	452 ± 37

HRT: hydraulic retention time; Q: flow rate; OLR: organic loading rate ^*^based on the sum of effective volume both ABR and BRS.

1 $$ \mathrm{t}=\frac{\mathrm{V}}{Q} $$

where V (L) is the void volume of the reactor, and Q (L/hour) is the design flow rate. Wastewater flow (L/day) expresses the design wastewater flow over a 24 hour period. Overall HRT indicates the sum of retention time both ABR and wetland system.

The inflow rates were fixed accordingly to 65, 80, 96, 114, 126 and 144 L/day for overall HRTs of 41, 33, 27, 23, 21 and 18 hours, respectively. The systems are operated at each retention time for 60 days, during which the effluent characteristics with an almost constant percentage reduction were achieved.

Additionally, the old plants were removed periodically and new plants were retained or allowed to grow during experimentations. In general, the roots of young plants can display greater ability to absorb impurities and release oxygen than old plants due to the age of the plants and tissues. Therefore, it is important to use healthy young plants for the more efficient contaminant removal [27]. Sodium bicarbonate is a buffer used to stabilize pH as per requirement in ABR.

### Wastewater sampling and analysis

Raw wastewater samples were collected from a residential source of WWRC, WRI, Iran, on a daily basis for performance evaluation. The samples from the inlet and outlet of the reactors was analyzed every 24 h at 10 am for pH, COD, SCOD, BOD_5_, TSS, TN, PO_4_-P and MPN of coliform bacteria according to the standard method [28]. In that order, the samples were merely filtered through Whatmann 42 before testing of SCOD and BOD_5_. The microbial population presented in the inlet and outlet, plant root surface, and Bio-rack surface were examined to determine the total viable count (TVC) using the pour plate technique [28]. The active attached biomass on the surface of Bio-racks was observed by using scanning electron microscope (SEM) as per the standard procedure [29]. Bacteria presented on the support matrix (Bio-racks) were merely isolated on nutrient agar medium and the Gram-negative bacterial identification was done using Mini API (BioMérieux, France).

### Botanical aspects

The morphological characteristics of *Phragmatis* sp. and *Typha* sp. in terms of the number of leaves, size of the leaves, number of roots, longest root, dry weight, and ash weight were analyzed every 15 days over a 3 month period throughout the six phases of the HRT evaluation to monitor morphological changes in the plants as per standard practice [30].

### Statistical analysis

The data of 365 consecutive days were analyzed to examine the performance of two integrated systems. Treatment efficiency was calculated as the percentage of removal for each parameter as follows:

2 $$ \mathrm{Removal}\ \mathrm{e}\mathrm{fficiency}\ \left(\%\right)=\frac{\mathrm{Ci}-\mathrm{C}\mathrm{e}}{\mathrm{Ci}}\times 100 $$

where C_i_ and C_e_ are the influent and effluent concentrations. Organic loading rates, OLR (g/day), were calculated by multiplying the discharge flow (L/day) and the organic concentrations (g/L). Relative growth rate of the plants is calculated using the following equation:

3 $$ \mathrm{Relative}\ \mathrm{growth}\ \mathrm{rate}\ \left(\%\right) = \frac{ \ln\ \mathrm{W}2 - \ln\ \mathrm{W}1}{\mathrm{t}2-\mathrm{t}1} \times 100 $$

where W_1_ and W_2_ are the dry weight of plants at times t_1_ and t_2_. Statistical analysis of the data to compare integrated systems on the organic load removal and morphological characteristics of plants growth were done through the variance (ANOVA) and Duncan test using SPSS software [31]. Performance differences were deemed to be significant if p <0.05.

## Results and discussions

This investigation discusses the possibility application of the Bio-rack wetland system as a post-treatment of anaerobic baffled reactor through single specimens of *Phragmites sp*. or *Typha sp*. for DEWATS. In the previous study, the performance of ABR throughout eight-compartment treating domestic wastewater was assessed, simulated, and optimum configuration was determined [9,10]. The ABR toward the treatment of domestic wastewater (750 mg COD/L) clearly showed a stable performance under different hydraulic retention times by having a high compartments number (more than 5). Therefore, the six-compartment ABR was selected in the present study to ensure an effective pre-treatment condition in treating domestic wastewater.

### Organic pollutant removal

Since, the constancy of any biological process thoroughly depends on the hydraulic and organic loading rates; the experiments were performed to estimate the satisfactory hydraulic retention time (HRT) of the whole integrated process of ABR followed by Bio-rack system using *Phragmites* sp. (ABR-BRSUP) and *Typha* sp. (ABR-BRSUT).

**Figure 2:**
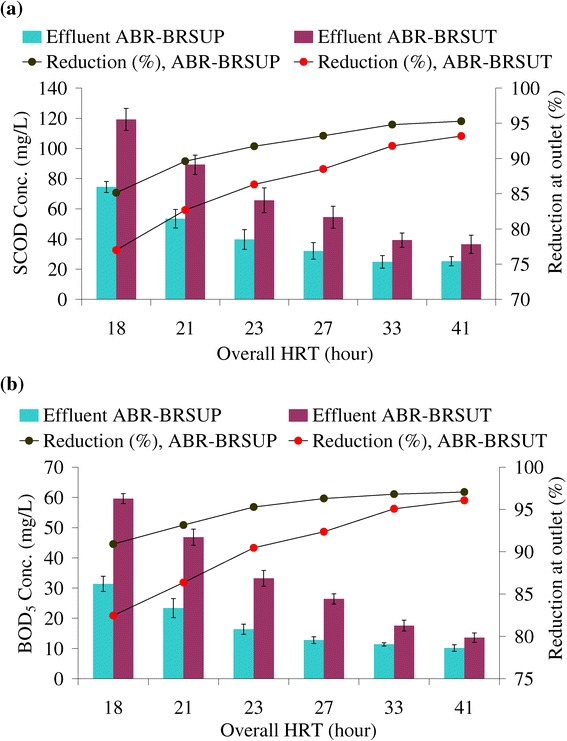
**Effluent** **(** **a** **)** **SCOD and** **(** **b** **)** **BOD**_**5**_ **concentrations with five days repetition through the integrated systems** **.**

The SCOD and BOD_5_ concentration of the treated effluent was below 60 and 30 mg/L at overall HRTs above 21 and 27 hours, in the treatment systems planted by *Phragmites* sp. and *Typha* sp., respectively (Figure 2). Likewise, the COD concentration was exceeded Irans’s permissible limit at below 21 (95 mg COD/L) and 27 (97 mg COD/L) hours HRTs. An ANOVA and Duncan test done on the performance data in terms of BOD_5_ removal indicate that the differences exist between the performances of these two processes; p-value was less than 0.05 merely at overall HRT of 21 and 18 hours (Table 2). The supremacy of the Bio-rack wetland vegetated with *Phragmites* sp. would be significant in lower retention times. Therefore, overall HRTs of 21 and 27 hours are considered to achieve a satisfactory organic removal performance in ABR-BRSUP and ABR-BRSUT, respectively.

**Table 2:** **Organic pollutant removal with five days repetition through the integrated systems**

**Overall HRT** **(** **hour** **)**	**BOD**_**5**_ **removed** **-** **g** **/** **day**	**BOD**_**5**_ **removed** **-** **g** **/** **day**	**P** **-** **Value**
	**(** **ABR**-**BRSUP** **)**	**(** **ABR**-**BRSUT** **)**	
41	21.85 ± 1.96	21.63 ± 1.90	0.486
33	27.71 ± 2.60	27.21 ± 2.50	0.398
27	31.95 ± 2.2	30.64 ± 2.23	0.102
23	37.85 ± 3.60	35.93 ± 3.56	0.062
21	40.14 ± 2.93	37.19 ± 2.80	0.016
18	45.21 ± 4.20	41.15 ± 4.25	0.007

HRT: hydraulic retention time;

ABR-BRSUP: anaerobic baffled reactor - bio-rack system using *Phragmites* sp.;

ABR-BRSUT: anaerobic baffled reactor - bio-rack system using *Typha* sp.

Many studies comparing planted systems over unplanted units have been conflicting results regarding the importance of plants in any phytoremediation process [32,33]. In the absence of the rhizomes of the plants, the micro-sites occupied by the wetland unit become anaerobic; lead to a lower rate of impurities elimination. The major advantage associated with the current integrated treatment systems is the presence of the Bio-rack wetland. The Bio-rack process planted with *Phragmites* sp and *Typha* sp. examined to offer, respectively, a lower HRT of 9.5 (COD: 77%, SCOD: 79%, BOD_5_: 84% reduction) and 12.5 (COD: 66%, SCOD: 69%, BOD_5_: 76% reduction) hours in polishing anaerobic effluent as compared to other relevant literatures [2,14,15]. Kaseva [14] reported average COD removal efficiencies of 56 & 61% at HRT of 1.96 and 1.99 days in the conventional constructed wetlands using *Phragmites* sp. and *Typha* sp., respectively. Mbuligwe [15] was determined the efficiency of conventional constructed wetlands using *Typha* sp. and *Colocasia* sp., respectively, by 79 & 75% COD reduction at 1.2 days HRT. The Bio-rack wetland used in this study also showed a higher removal of organic pollutant compared to the duckweed system, in which 54 & 58% COD, 63 & 64% SCOD and 21 & 63% BOD_5_ reduction could be accomplished at 10 and 15 days HRT, respectively [2]. It can be verified that wetland type, efficient oxygen diffusion, and the high accumulation of the attached bacterial populations on the surface of support matrix are the major factors for high level of removal efficiency in the Bio-rack process.

The Bio-rack wetland with *Phragmites* sp. exhibits a higher rate of pollutant reduction owing to the extensive root system of the plant which is responsible for desirable rate of the oxygen transfer efficiency, and greater microbial mass contribution in the treatment unit. However, there was a little difference in the removal of organic pollutant at longer retention times during the operation of both treatment processes. The visual observation showed that the *Phragmites* sp. tends to develop a wonderfully root system which occupies thoroughly over the depth of the treatment unit. *Typha* sp. appears to have a shallower root system.

### Dissolve oxygen silhouette in the reactor

**Figure 3:**
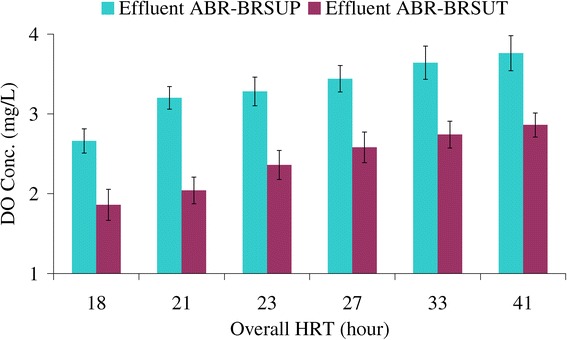
**Mean DO values of the processes effluent with five days repetition.**

Adequate dissolved oxygen required for bacterial oxidation is relatively inefficient by a transfer process of atmospheric diffusion, and in the Bio-rack wetland the transfer is likely to be smaller since the dense plant cover reduces surface gas-exchange. Vegetation plays a significant role in transferring oxygen via aerenchyma tissue [32]. The dissolved oxygen inside the Bio-rack cells was estimated to be an average of 3.2 and 2.6 mg/L O_2_ at overall HRTs of 21 and 27 h in ABR-BRSUP and ABR-BRSUT, respectively (Figure 3). The results suggest that the mechanism of aerobic respiration is a key microbial population in the Bio-rack process due to high rate of oxygen transfer efficiency through the roots. The aerobic bacteria are fast growing microflora causing a greater rate of organic elimination compared to anaerobic microorganism. The higher level of dissolved oxygen in the Bio-rack unit planted with *Phragmites* sp. probably can be explained by type of plant having an extensive root system; allows oxygenation to occur from the roots through the entire depth of the treatment unit.

### Treatment performance

A comprehensive evaluation of other parameters was conducted at reasonable overall HRTs of 21 and 27 h (Table 3). Regarding to the results, it can be concluded that high organic loads of BOD_5_ and TSS of influent can be efficiently alleviated in ABR as a pretreatment of the Bio-rack wetlands. The data evaluated in the previously reported literature, likewise, specified a possible need of a longer retention period in the Bio-rack wetlands if they use as a standalone method in treating domestic wastewater with high organic pollutant concentrations (COD > 440 mg/L, BOD_5_ > 238 mg/L), whenever plants respond positively [21]. Therefore, the appropriateness of the ABR process for primary sanitation can be remarkable to improve the performance of the phytoremediation systems; meanwhile it can also decrease choking and clogging problems associated with quality variations of the influent in the conventional wetland processes. This can be implied by standard deviations shown in Table 3.

**Table 3:** **Summary of effluents characteristic with five days repetition from two integrated treatment systems**

Parameters*	**ABR**-**BRSUP** **(** **overall HRT of 21 hours** **)**				**ABR**-**BRSUT** **(** **overall HRT of 27 hours** **)**			
	**Inlet**	**Outlet at ABR**	**Outlet at BRSUP**	**Overall reduction** **(%)**	**Inlet**	**Outlet at ABR**	**Outlet at BRSUT**	**Overall reduction** **(%)**
pH	7.45 ± 0.09	7.10 ± 0.05	7.19 ± 0.04	-	7.50 ± 0.10	7.15 ± 0.06	7.29 ± 0.05	-
DO	0.16 ± 0.05	-	3.2 ± 0.14	-	0.18 ± 0.06	-	2.6 ± 0.20	-
COD	736 ± 51.28	420.4 ± 30.96	95.2 ± 2.28	87.1	713 ± 21.40	290 ± 17.04	97.4 ± 1.52	86.3
SCOD	515 ± 42.51	256 ± 25.26	53.4 ± 6.12	89.6	473 ± 24	173.8 ± 26.25	54.4 ± 7.85	88.5
BOD_5_	342 ± 24.36	150 ± 12.32	23.4 ± 3.13	93.2	345.6 ± 22	110 ± 8.12	26.4 ± 1.67	92.4
TSS	523.4 ± 48.22	245 ± 42.66	61 ± 11.71	88.3	521 ± 47	237.2 ± 28.62	71.2 ± 7.33	86.3
TN	67.97 ± 3.88	56.31 ± 5.17	14.02 ± 0.64	79.4	63.45 ± 5.72	51.25 ± 4.61	14.41 ± 0.32	77.3
PO_4_-P	13.65 ± 0.65	20.26 ± 1.82	10.73 ± 1.16	21.4	15.30 ± 0.43	22.69 ± 2.25	13.13 ± 1.04	14.2

*All analytical values except pH are based on mg/L.

Both integrated systems can also be a viable option to reduce nutrient levels by more than 75% TN and 14% PO_4_-P. Dissolved oxygen concentration would seem to be more than 2.5 mg/L in the wetland units. *Phragmites* sp. has a great potential distribution of roots enhanced nutrient uptake by the root segments in their favorable environment. Literature based evaluation confirms that the present processes potentially have a higher nutrient reduction of 40-58% TN compared to the anaerobic baffled reactor as a standalone solution [2]. It would emphasize that the application of the Bio-rack wetlands, as a post treatment of ABR can effectively improve the quality of treated domestic wastewater to be discharged to surface waters in rural areas.

The suspended solids are removed throughout the integrated systems via sedimentation and filtration phenomena. The efficiency of nutrient (N,P) removal can be explained adequately by the nitrification/denitrification of rhizoremediation process and plant uptake route. Nitrogen removal is rather high for both integrated systems. Phosphorus removal is notoriously poor in any constructed wetland practices [32]. The anaerobic systems typically increase the soluble amount of phosphate while can hardly be removed efficiently through the phytoremediation techniques. The greater percentage reduction of phosphorus can be attained by incorporating the advantages of using chemical divalent cations as a result of superior exchange reaction mechanism. Furthermore, the nutrient elimination would be succeeded by frequent harvesting of plants based on climate condition.

### Microbial investigation

**Figure 4:**
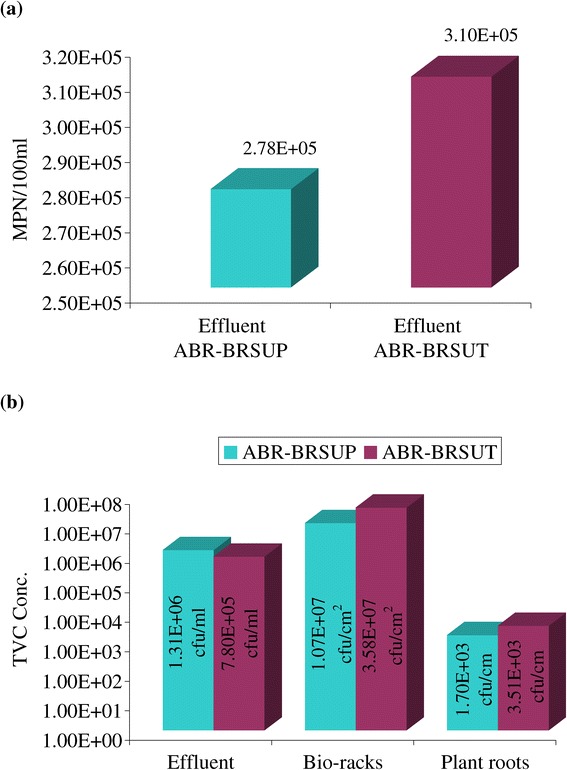
**Mean** **(** **a** **)** **total coliform and** **(** **b** **)** **total viable count values through the integrated systems.**

The MPN of total coliform and TVC concentrations at the outlet of two integrated processes at overall HRTs of 21 and 27 h are shown in Figure 4. The average reduction of these parameters was observed approximately 99% at the outlet end of the processes. In wetland units, there is a reduction of the TVC level at the outlet, and biological mass is getting attached to the surface of the Bio-racks. This could be phenomena in the initial stage of operation. At steady state condition, there will be uniform growth on the Bio-rack surfaces, and biomass will slough and come out with the flowing water.

The microorganisms create a sort of natural polymer responsible for their adhesion to develop the biofilm and bio-oxidation mechanism [34]. The support matrix (Bio-rack) provides the evidence of enormous TVC concentrations (Figure 4b). TVC concentrations were also found to be high both on the root surface of *Phragmites* sp. and *Typha* sp. It should be realized that *Phragmites* sp. with its extensive root system enhanced bacterial population throughout the Bio-rack unit. In the Bio-rack wetland, the vicinity of the support matrix, stems and roots of the plants are a preferred environment for many micro-organisms to attach and to degrade pollutants in the phytoremediation process.

**Figure 5:**
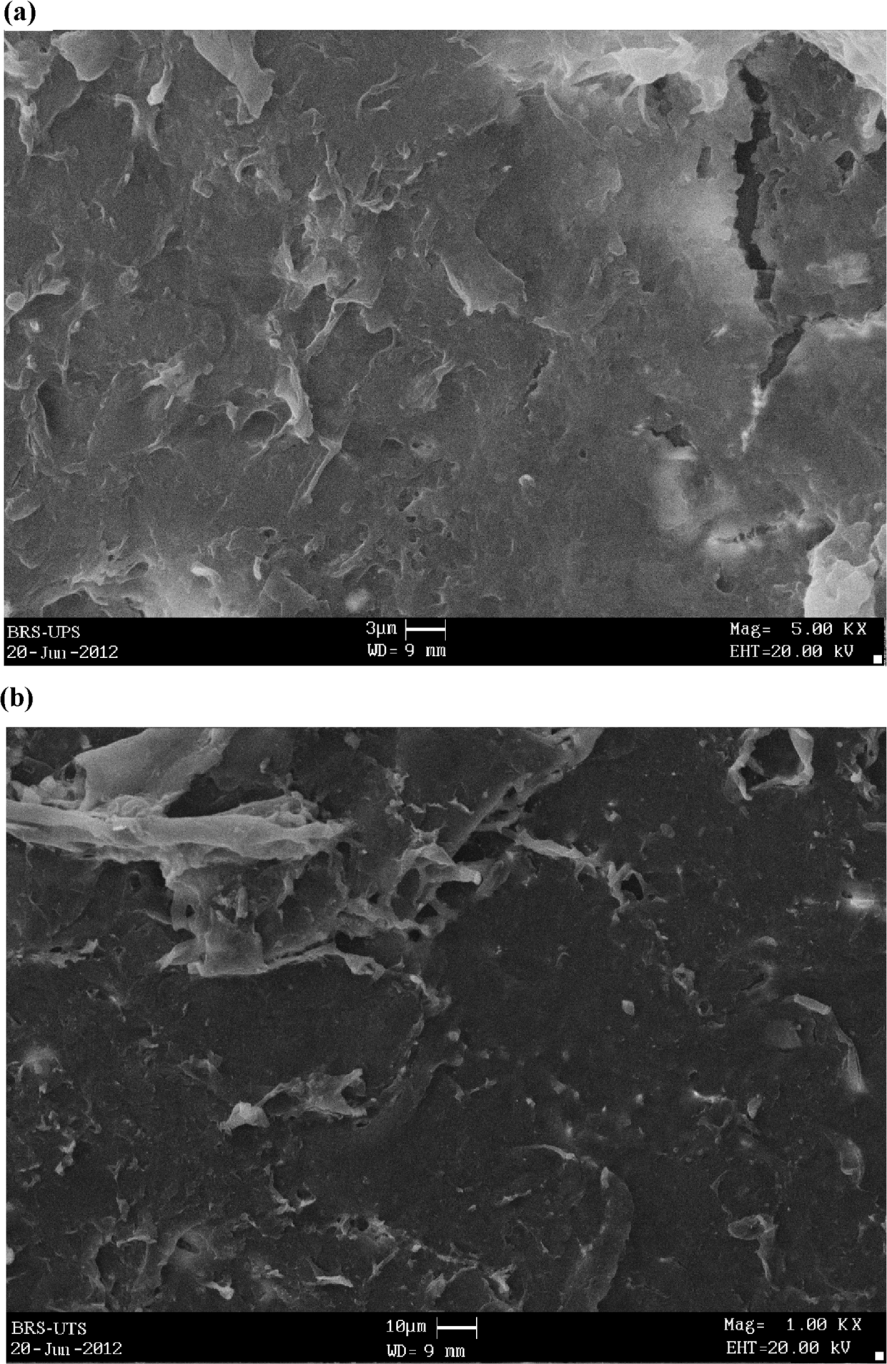
**SEM photographs of the biofilm** **-** **forming bacteria on the surface of Bio** **-** **racks in** **(** **a** **)** **ABR**-**BRSUP and** **(** **b** **)** **ABR** **-** **BRSUT** **.**

The biofilm adhesion in the context of bacterial formation was visualized by scanning electron microscope (SEM). The microscopic examinations revealed the bulk of the biomass concentration on the Bio-rack surface media in both ABR-BRSUP and ABR-BRSUT (Figure 5). The biofilm, which were grown on the surface of the Bio-racks, promote somewhat a denser morphology in ABR-BRSUP (overall HRT of 21 hours) compared to ABR-BRSUT (overall HRT of 27 hours). This can be demonstrated by a high flow condition at a low retention time where the viscoelastic nature of extracellular biopolymers holding the microcolonies together. In ABR-BRSUT, the morphology of biofilm grown at a low flow discharge exhibit a nearly lower dense and microcolonies are tapered. The high flow condition can also result in the detachment of biofilm, reduce the biofilm bacteria quantity, and increase of effluent bacteria population as a result of a sloughing process. This can be evident from the results on TVC (Figure 4b). Similar phenomena were also reported in the relevant literature [35]. The amount of biofilm formed on the surface of the Bio-rack was estimated to be 7 to 8 and 8 to 9 g/L in BRSUP and BRSUT, respectively.

Correspondingly, the profiling of microflora population on the surface of Bio-racks can show the presence of approximately 12 bacterial strains grown on nutrient agar medium i.e. *Acinetobacter lwoffii*, *Aeromonas sobria*, *Escherichia coli*, *Pseudomonas stutzeri*, *Aeromonas hydrophila*, *Citrobacter koseri*, *Acienetobacter johnsonni*, *Entrobacter intermedius*, *Alcaligenes faecalis*, *Pantoea sp*., *Pseudomonas putida*, *Acinetobacter baumanni*; this could verify the results of previous work [21]. It should be noted that there is a possibility of other microbial population, which could not be detected in a nutrient agar medium.

### Plant morphological study

A study of the morphological aspects of the vegetation clearly indicated that anaerobically treated domestic sewage had no detrimental effect on the plant morphology, and the yield of the plants was increased (Table 4). In this study, anaerobically treated effluents were enriched with nutrients, particularly nitrogen (Table 3). The absorption and assimilation of nutrients by plants and the natural pH of the treated effluents may be the reasons for positive influence on the plant growth. Relative growth rate of plants also showed that *Phragmites* sp. would be more resilient to grow (3.92%) in the pretreated anaerobic effluent than *Typha* sp. (0.90%). The statistical analysis also indicated that there is a significant difference between plants in their growth (p < 0.05), except the longest root which found not significantly different (p > 0.05). These findings suggest that the *Phragmites* sp. which has a higher mass growth rate can contribute most to phytoremediation process for pollutant removal within a wetland unit (Table 3). The wetland macrophytes can adapt morphologically to grow in a water-saturated substrate through aerenchyma [36].

**Table 4:** **Morphology characteristics of the plants at initial step and after 15 days operation** (**n** = **5**)

**Parameter**	**BRSUP**		**BRSUT**		**P** **-** **Value**
	**Initial**	**After 15 days**	**Initial**	**After 15 days**	
Number of leaves	6.2 ± 1.8	10.4 ± 1.14	8.8 ± 1.3	12 ± 1.6	0.0393
Size of leaves (mm^2^)	24.3 ± 2.75	26.65 ± 2.17	96.02 ± 22	140.05 ± 20	0.0001
Number of roots	9.8 ± 1.5	28.2 ± 5.8	45.4 ± 17.52	94.6 ± 18.25	0.0005
Longest root (mm)	12.7 ± 1.43	16.42 ± 1.05	19.34 ± 4.2	22.64 ± 5	0.0670
Dry weight (g/plant)	0.65 ± 0.5	1.17 ± 0.6	28.39 ± 1.7	32.49 ± 1.3	0.0014
Ash weight (g/plant)	0.035 ± 0.006	0.114 ± 0.05	3.018 ± 0.42	3.91 ± 0.2	0.0002

### Pre-estimation of capital cost

A pre-estimation of economic analysis has been done for 1 m^3^/day of sewage inflow based on the results generated by bench scale trials (Table 5). The result of this analysis suggested that the application of the ABR-BRSUP process could lead to 24% less land space than the ABR-BRSUT; making the phytoremediation process more sustainable for DEWATS. The total capital investment of ABR-BRSUP and ABR-BRSUT for an estimated capacity of 1 m^3^/day sewage is $US 90, and $US 120, respectively. The purpose of this pre-estimation was merely to verify the advantages within the present integrated treatment systems. However, both processes can provide added benefits of an overall low HRT and high pollutant removal efficiency compared to other reported literatures [2,14,15]. The low HRT in the present integrated systems reduces the land space requirement; makes the treatment method more valuable. A detailed cost analysis of these processes needs to be performed in subsequent pilot and field experiments.

**Table 5:** **Pre**-**estimated capital cost of integrated treatment systems** (**Q** =**1m** ^**3**^/**d**, **COD** <**100 mg**/**L**, **and BOD**_**5**_ < **30 mg**/**L**)

**Item**	**ABR**-**BRSUP**		**ABR**-**BRSUT**	
	**ABR**	**BRSUP**	**ABR**	**BRSUT**
Volume (m^3^/m^3^_inflow_)	0.48	0.41	0.63	0.54
Cost				
US$/m^3^_inflow_	30	60	40	80
US$/kg BOD_5_ removed	155	480	167	950
Total cost				
US$/m^3^_inflow_	90		120	
US$/kg BOD_5_ removed	635		1117	

ABR: anaerobic baffled reactor; BRSUP: bio-rack system using *Phragmites* sp.; BRSUT: bio-rack system using *Typha* sp.

It can be summarized that the anaerobic baffled reactor followed by Bio-rack wetland can accomplish high rate organic degradation; however, the application of *Phragmites* sp. can make the treatment process partially more concise and reliable. Yet, the effluent of this process requires disinfection before discharging into the river. In addition, the effluent can also be soil infiltrated in soak pit, leach fields and mounds or else discharged underground at soil depths.

## Conclusions

The studies highlight the performance of an anaerobic baffled reactor followed by Bio-rack wetland planted with *Phragmites* sp. and *Typha* sp. for decentralized treatment of domestic wastewater. These processes are characterized by other integrated treatment practices, where conventional wetland systems have provided in case further polishing of anaerobic effluent and in lieu, a support matrix (Bio-rack) is provided to enrich the aerobic bacteria in the form of biofilm within wetland unit. They eliminate possible limitations of ABR and Bio-rack wetland as standalone solutions, respectively, relating to low nutrient elimination and high organic load restriction. The integrated system planted with *Phragmites* sp. has low space requirement, low capital investment, and most importantly, high degradation of organic pollutants, which was capable of achieving COD, SCOD, and BOD_5_ removal as high as 87%, 90% and 93%, respectively, at an overall HRT of 21 h. The integrated process using *Typha* sp. also showed stable and consistent performance in degradation of organic pollutants, where it was possible to remove 86% COD, 89% SCOD, and 92% BOD_5_ at an overall HRT of 27 h. Both of these integrated processes are effective for nitrogen removal (77-79%) whereas they are less efficient in removing phosphorus (14 - 21%); divalent cation materials can be incorporated. The relative growth rate could make *Phragmites* (3.92%) a good species for phytoremediation activities than *Typha* sp. (0.90%). *Phragmites* sp. has adapted its root system to extend over the depth of the wetland unit, resulting in higher oxygen transfer efficiency through the roots, better nutrient uptake, and larger surface area for aerobic microbial biofilm to adhere and growth compared to *Typha* sp. This is a principal function of *Phragmites* sp. that can potentially increase pollutant reduction in the treatment unit (p < 0.05). It can be concluded that present integrated processes (especially by using *Phragmites* sp.) can be an ideal technology for DEWATS; however, this calls for detail pilot scale and field studies.
